# Sparse Fusion Imaging for a Moving Target in T/R-R Configuration

**DOI:** 10.3390/s140610664

**Published:** 2014-06-17

**Authors:** Shougang Chai, Weidong Chen, Chang Chen

**Affiliations:** Key Laboratory of Electromagnetic Space Information, Chinese Academy of Sciences, University of Science and Technology of China, Hefei 230027, China; E-Mails: chshg@mail.ustc.edu.cn (S.C.); chench@ustc.edu.cn (C.C.)

**Keywords:** sparse imaging, fusion imaging, T/R-R configuration, ISAR, bistatic ISAR, resolution improvement, moving target imaging

## Abstract

For high resolution imaging of a non-cooperative moving target, this paper proposes a sparse fusion imaging method. The imaging system contains two radar stations, which are separated by a certain bistatic angle and configured in a transmitter/receiver-receiver (T/R-R) manner. Consequently, two synthetic apertures are obtained at the same time from different aspect angles. By coherently fusing the echoes of the two radars, a virtual aperture spanned by these two sub-apertures can be constructed, which is larger than either of the sub-apertures; thus, the cross-range resolution of the image is enhanced. Moreover, the fusion of the echoes is realized by exploiting the sparse scattering property of the target. Then, based on the maximum a *posteriori* (MAP) criterion, the T/R-R fusion imaging problem is converted into a sparse signal recovery problem with unknown parameters. Finally, it is solved in an iterative manner, which contains two steps, *i.e.*, sparse imaging and parameter estimation. Simulation results show that the proposed sparse fusion imaging method can improve the cross-range resolution significantly compared to inverse synthetic aperture radar (ISAR) within the same coherent processing interval (CPI).

## 1. Introduction

Microwave radar imaging is one of the major techniques for non-cooperative moving target recognition (NCTR). Compared to optical or infrared imaging, it has the ability to work under all weather, all time and long-range conditions. Therefore, it plays an important role in both defense and civilian applications [1–4].

Inverse synthetic aperture radar (ISAR) is the traditional microwave imaging method for a moving target [5–8]. In ISAR imaging, the target is mapped onto a slant range and cross-range plane, where the slant range resolution is determined by the bandwidth of the transmitted signal and the cross-range resolution is obtained by exploiting the motion of the target. Usually, the motion is decomposed into a translational and a rotational component [5]. After the translational motion is compensated, the rotational motion forms a synthetic aperture with size being Δ*θ* = *ωT*, where *ω* is the rotational angular speed and *T* is the coherent processing interval (CPI). Since the cross-range resolution is inversely proportional to Δ*θ* and *ω* is determined by the non-cooperative motion of the target, a long CPI time is required in order to obtain high-cross resolution. However, a long CPI time will make the motion compensation difficult [5–8]. As a variant of monostatic ISAR, bistatic ISAR (B-ISAR) has also been studied recently [9–12], where the transmitter and the receiver are separated by a bistatic angle. Although it can overcome some geometry limitations existing in monostatic ISAR, high cross-range resolution also cannot be guaranteed, due to the same reasons in ISAR.

**Figure 1.**
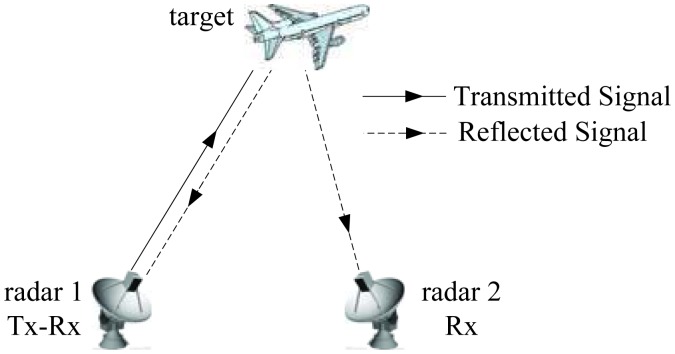
Imaging configuration.

For the purpose of high resolution imaging with short CPI time, this paper proposes a sparse fusion imaging method by combining the monostatic ISAR and B-ISAR together. As shown in Figure 1, the imaging system consists of only two radar stations, which are separated by a certain bistatic angle and configured in a transmitter/receiver-receiver (T/R-R) manner. Consequently, two sets of echoes are received by Radar 1 and Radar 2, each of which corresponds to a sub-aperture located at different aspect angles. The interval between the two sub-apertures is equal to half the bistatic angle [9,10]. Under the condition of short CPI, usually, the half bistatic angle is larger than the sizes of the sub-apertures; in other words, there is an aperture gap between them. Therefore, by coherently fusing the received data, a virtual aperture spanned by these two sub-apertures can be constructed, which is much larger than either of the sub-apertures. As a result, the cross-range resolution will be improved significantly.

In the echoes fusion procedure, due to relatively large aperture gap and short CPI, traditional Fourier-based sub-aperture fusion methods will suffer from high side lobes, and the spectrum estimation-based gaped-data fusion methods [13,14] will also get performance degradation. In addition, three parameters (the rotational speeds of target relative to Radar 1 and Radar 2 and the bistatic angle) are required in order to fuse the echoes coherently. However, it is difficult to obtain accurate estimations directly from the return signals, due to short CPI time [15–17]. Here, we propose a new fusion imaging method by exploiting the sparse scattering property of the target. Based on the maximum *a posteriori* (MAP) criterion, the data fusion and the parameter estimation are combined together and converted into a joint optimization problem. Then, it is solved in an iterative manner by alternating two steps: sparse imaging and parameter estimation, where the first step is a sparse signal recovery (SSR) problem [18–22] and the second step can be solved by a linear search method.

The remainder of the paper is organized as follows: Section 2 gives the imaging geometry and the return signal models. The basic idea of the imaging system and the sparse fusion imaging method are presented in Section 3. Then, the imaging algorithm is described in Section 4, and the numerical results are shown in Section 5 to validate the proposed method. Finally, Section 6 is the conclusion.

## 2. Return Signal Modeling

**Figure 2.**
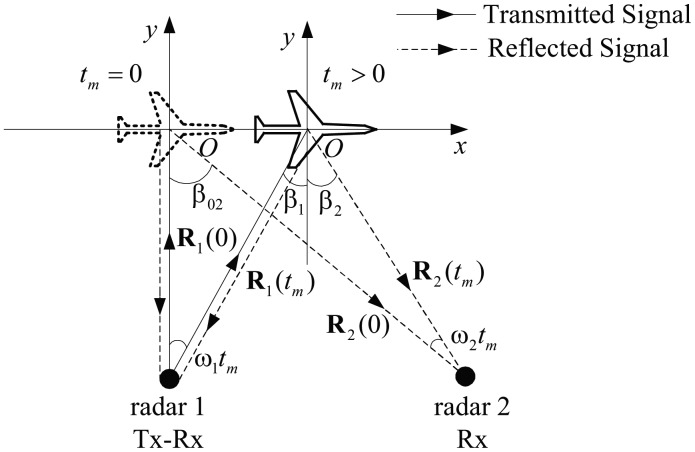
Imaging geometry.

The imaging geometry is shown in Figure 2. Radar 1 transmits the signal and then receives the reflected signal, while Radar 2 works only as a receiver. For simplicity, we assume that the trajectory of the target is on the plane spanned by the line of sights (LOSs) of Radar 1 and Radar 2 during the short CPI time. It is worth noticing that the return signal model and imaging method introduced in this paper can be easily generalized to the case where the motion of the target is not parallel to that plane [23,24]. Let *t_m_* ∈ [−*T_m_*/2, *T_m_*/2] be the slow time (snapshot time) and *T_m_* be the CPI. Figure 2 shows two instants of the target at *t_m_* = 0 and *t_m_* > 0, where the target is represented with a dashed line and a solid line, respectively, and **R**_1_(*t_m_*) and **R**_2_(*t_m_*) are the LOSs of Radar 1 and Radar 2.

The space coordinate *xOy* is established with respect to the target by fixing the coordinate origin on the center of the target and setting **R**_1_(0) (the LOS of Radar 1 at the snapshot time *t_m_* = 0) as the *y*-axis. According to the manner of ISAR, we define the *y*-axis as the slant range direction and the *x*-axis as the cross-range direction.

Let *β*_1_(*t_m_*) and *β*_2_(*t_m_*) be the angles of the two radars' LOSs with respect to the *y*-axis. Referring to our previous study [9], we treat the motions of the target relative to Radar 1 and Radar 2 separately and decompose each of the relative motions into a translational and a rotational component. During the short CPI time, the rotational motions of the target relative to Radar 1 and Radar 2 are approximately uniform rotations. Suppose the two rotational speeds are *ω*_1_ and *ω*_2_, respectively; then *β*_1_(*t_m_*) and *β*_2_(*t_m_*) can be expressed as follows:

(1) $$\begin{array}{cc} \beta_{1}\left(t_{m}\right)=\omega_{1}t_{m}, & \beta_{2}\left(t_{m}\right)=\beta_{02}-\omega_{2}t_{m} \end{array}$$

where *β*_02_ is the abbreviation of *β*_2_(0), which is the bistatic angle of Radar 2 at *t_m_* = 0.

First we give the return signal model of Radar 2, because that of Radar 1 is the special case of Radar 2. According to the high-frequency scattering mechanism, the target can be modeled as a set of dominant point scatterers [25–30]. Therefore, for the *i*^th^ point scatterer located at r*_i_* = (*x_i_*, *y_i_*), under the condition of far field approximation, we can obtain the delay time at snapshot time *t_m_* as Equation (2):

(2) $$\tau_{2}\left(t_{m};x_{i};y_{i}\right)\approx \frac{1}{c}\left[\left|{\text{R}}_{1}\left(t_{m}\right)\right|+\left|{\text{R}}_{2}\left(t_{m}\right)\right|+y_{i}-x_{i}\sin\beta_{02}+y_{i}\cos\beta_{02}+x_{i}\omega_{1}t_{m}+x_{i}\omega_{2}t_{m}\cos\beta_{02}+y_{i}\omega_{2}t_{m}\sin\beta_{02}\right]$$

According to linear scattering theory, the return signal of Radar 2 is the superposition of the signals reflected from all of the scatterers. After matched filtering and translational motion compensation, the return signal of Radar 2 can be written as:

(3) $$s_{2}\left(\hat{t},t_{m}\right)=\underset{i}{\text{∑}}a_{2}\left(x_{i},y_{i},\hat{t},t_{m}\right)\sigma_{i}+e_{2}\left(\hat{t},t_{m}\right)$$

where *tˆ* ∈ [0, *T_p_*] is the fast time, *T_p_* is the pulse repetition period, ***σ**_i_* is the scattering coefficient of the *i*^th^ point scatterer, *e*_2_ is the additive white Gaussian noise (AWGN) and *a*_2_ is:

(4) $$a_{2}\left(x_{i},y_{i},\hat{t},t_{m}\right)=\text{sinc}\left\{\pi B\left[\hat{t}-\frac{1}{c}\left(y_{i}-x_{i}\sin\beta_{02}+y_{i}\cos\beta_{02}\right)\right]\right\}\times e^{-\text{j}2\pi \frac{f_{0}}{c}\left(y_{i}-x_{i}\sin\beta_{02}+y_{i}\cos\beta_{02}+x_{i}\omega_{1}t_{m}+x_{i}\omega_{2}t_{m}\cos\beta_{02}+y_{i}\omega_{2}t_{m}\sin\beta_{02}\right)}$$

where *f*_0_ is the carrier frequency, *B* is the bandwidth and the migration through the range resolution cell is not considered.

Then, we translate the return signal into its discrete form. Uniformly dividing the imaging scene into *K* × *L* spatial positions along *x* and *y* directions with grid sizes being *dx* and *dy*, respectively. As a result, the scattering coefficients of the imaging area can be expressed as a matrix 𝚵 = [***σ**_k,l_*]*_K_*_×_*_L_*. Suppose that there are *N* samples in fast time and *M* samples in slow time; we use *n* to express the discretized fast time and *m* to represent the discretized snapshot time. Then, the return signal of Radar 2 can be expressed as an *N* x *M* matrix:

(5) $$\begin{array}{ccc} S_{2}=\overset{K}{\underset{k=1}{\text{∑}}}\overset{L}{\underset{l=1}{\text{∑}}}A_{2}^{k,l}\sigma_{k,l}+E_{2} & k=1,\ldots ,K, & l=1,\ldots ,L \end{array}$$

where ${\text{A}}_{2}^{k,l}={\left[a_{2,n,m}^{k,l}\right]}_{N\times M}$ is the observing matrix with respect to the point scatterer located at (*k*, *l*) and **E**_2_ is the *N* × *M* noise matrix.

Next, we express Equation (5) in its vector form: First let **s**_2_ = vec(**S**_2_), where vec(·) refers to the vectorization operation (*i.e.*, stacking the columns of a matrix on top of each other) [22]. Then, define the observing matrix of Radar 2 as:

(6) $$A_{2}=\left[\text{vec}\left(A_{2}^{1,1}\right),\text{vec}\left(A_{2}^{2,1}\right),\ldots ,\text{vec}\left(A_{2}^{K,1}\right),\text{vec}\left(A_{2}^{1,2}\right),\text{vec}\left(A_{2}^{2,2}\right),\ldots ,\text{vec}\left(A_{2}^{K,2}\right),\ldots ,\text{vec}\left(A_{2}^{1,L}\right),\text{vec}\left(A_{2}^{2,L}\right),\ldots ,\text{vec}\left(A_{2}^{K,L}\right)\right]$$

Furthermore, let ***σ*** = vec (𝚵) and **e**_2_ = vec(**E**_2_). In this way, the return signal of Radar 2 can be expressed in the following compact form:

(7) $${\text{s}}_{2}={\text{A}}_{2}\text{σ}+e_{2}$$

The return signal of Radar 1 is the special case of that of Radar 2. Let *β*_02_ = 0 and replace *ω*_2_ with *ω*_1_, we can obtain:

(8) $${\text{s}}_{1}={\text{A}}_{1}\text{σ}+{\text{e}}_{1}$$

where **A**_1_ is the observing matrix of Radar 1 and **e**_1_ is an AWGN vector.

## 3. Sparse Fusion Imaging in T/R-R Configuration

**Figure 3.**
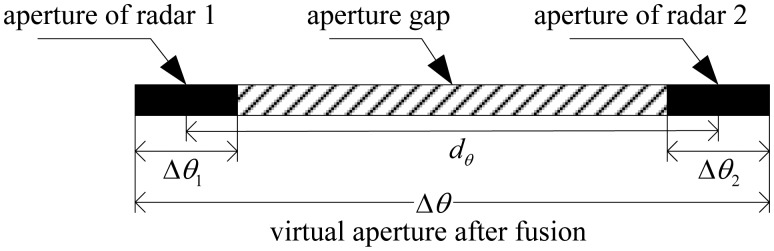
Schematic diagram of the synthetic apertures of the transmitter/receiver-receiver (T/R-R) imaging system.

Figure 3 shows the basic idea of the proposed imaging method from the perspective of synthetic aperture, where Δ*θ*_1_ and Δ*θ*_2_ are the apertures of Radar 1 and Radar 2, respectively. In a T/R-R imaging system, the two radars receive return signals from different viewing directions, so Δ*θ*_1_ and Δ*θ*_2_ are obtained at the same time and located at different aspect angles. Suppose the interval between them is *d _θ_*; then, according to the theory of ISAR and B-ISAR [5,9−11], we can obtain that:

(9) $$\begin{array}{l} \Delta \theta_{1}=T_{m}\omega_{1}\\ \Delta \theta_{2}=T_{m}\left(\omega_{1}+\omega_{2}\cos\beta_{02}\right)/2\\ \Delta \theta_{1}=\beta_{02}/2 \end{array}$$

As is well known, large synthetic aperture is required in order to improve the cross-range resolution of the image. In this paper, we will try to construct the large virtual aperture Δ*θ* by coherently fusing the echoes **s**_1_ and **s**_2_. As shown in Figure 3, this virtual aperture spanned by the sub-apertures Δ*θ*_1_ and Δ*θ*_2_ has a size of:

(10) $$\Delta \theta =\left(\Delta \theta_{1}+\Delta \theta_{2}\right)/2+d_{\theta }$$

Because *d_θ_* = *β*_02_/2 > 0, Δ*θ* is larger than either of Δ*θ*_1_ and Δ*θ*_2_. Specifically, we consider two cases:

(1) when 0 < *d_θ_* ≤ (Δ*θ*_1_ + Δ*θ*_2_) /2, Δ*θ*_1_ and Δ*θ*_2_ will overlap with each other partially, and we can obtain that (Δ*θ*_1_ + Δ*θ*_2_) /2 < Δ*θ* ≤ (Δ*θ*_1_ + Δ*θ*_2_). In this case, the cross-range resolution can be improved by fusing **s**_1_ and s_2_, but the improvement factor will be less than two.
(2) when *d_θ_* > (Δ*θ*_1_ + Δ*θ*_2_) /2, the two sub-apertures will be separated without overlapping; in other words, there will be an aperture gap between them, as shown in Figure 3. Especially under the condition of short CPI and a relatively large bistatic angle, the aperture gap will be much larger than the sub-apertures themselves. As a result, Δ*θ* will become much larger than (Δ*θ*_1_ + Δ*θ*_2_). If this virtual large aperture can be obtained, then the cross-range resolution will be improved significantly. Furthermore, note that in order to obtain Δ*θ* by coherently fusing s_1_ and s_2_, the scattering properties of the target with respect to the LOSs of Radar 1 and Radar 2 must be approximately the same. Therefore, generally, the bistatic angle *β*_02_ is constrained to be less than 10° [31].

For the first case, the spanned aperture can be obtained by traditional sub-aperture fusion methods [23,24]. This paper will focus on the second case, which is more likely to occur in T/R-R fusion imaging for non-cooperative moving target with short CPI. Above all, based on the return signal models (7) and (8), we combine them together and construct a fusion model as follows:

(11) s=Aσ+e

where:

(12) $$s=\left(\begin{array}{c} s_{1}\\ s_{2} \end{array}\right),\;A=\left(\begin{array}{c} A_{1}\\ A_{2} \end{array}\right),\;e=\left(\begin{array}{c} e_{1}\\ e_{2} \end{array}\right)$$

**s** is the combined return signal vector; **A** is the corresponding observing matrix, and e is the noise vector. Suppose **e**_1_ and **e**_2_ are independent and identically distributed AWGN vectors; then, e is also an AWGN vector with the mean being zero and the covariance matrix being *η***I**, where *η* is the power of the noise, which is an unknown parameter to be estimated.

According to Equations (4) and (11), **A** is determined by three unknown parameters *ω*_1_, *ω*_2_ and *β*_02_. To fuse **s**_1_ and **s**_2_ coherently, these three parameters must be estimated. However, due to the short CPI time, it is difficult to obtain accurate estimations of the parameters directly from the return signals in advance [15–17]. In this paper, we combine the fusion imaging procedure, as well as parameter estimation together and treat the scattering coefficients vector ***σ*** also as an unknown parameter. Then, there are a total of five unknown parameters in Equation (11), *i.e.*, ***σ***, *η*, *ω*_1_, *ω*_2_ and *β*_02_. Therefore, the fusion imaging problem (11) can be seen as a multi-parameters estimation problem. Assuming that the parameters ***σ***, *η*, *ω*_1_, *ω*_2_ and *β*_02_ are uncorrelated, then according to the MAP criterion [32], we can obtain these parameters by the following joint optimization problem:

(13) $$\begin{array}{ll} \left(\hat{\sigma },\hat{\eta },{\hat{\omega }}_{1},{\hat{\omega }}_{2},{\hat{\beta }}_{02}\right) & =\arg\underset{\sigma ,\eta ,\omega_{1},\omega_{2},\beta_{02}}{\max}f\left(\sigma ,\eta ,\omega_{1},\omega_{2},\beta_{02}|\text{s}\right)\\ & \propto \arg\underset{\sigma ,\eta ,\omega_{1},\omega_{2},\beta_{02}}{\max}f\left(\text{s}|\sigma ,\eta ,\omega_{1},\omega_{2},\beta_{02}\right)f\;\left(\sigma \right)\;f\;\left(\eta \right)\;f\;\left(\omega_{1}\right)\;f\;(\omega_{2})\;f\left(\beta_{02}\right) \end{array}$$

where ***σ**ˆ*, *ηˆ*, *ωˆ*_1_, *ωˆ*_2_, *βˆ*_02_ are the estimations of ***σ***, *η*, *ω*_1_, *ω*_2_ and *β*_02_, respectively.

Because e is an AWGN vector, the conditional probability density function of s is:

(14) $$f\left(\text{s}|\sigma ,\eta ,\omega_{1},\omega_{2},\beta_{02}\right)=CN\left(\text{A}\sigma ,\eta \text{I}\right)$$

where 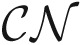 represents the complex Gaussian distribution.

As there is no *a priori* knowledge about *η*, *ω*_1_, *ω*_2_ and *β*_02_, thus we use the non-informative prior [33] as their distributions according to Bayesian statistics:

(15) $$\begin{array}{cc} f\left(\eta \right)f\left(\omega_{1}\right)f\left(\omega_{2}\right)f\left(\beta_{02}\right)\propto 1, & \eta \in \left[0,\eta_{\max}\right], \end{array}\omega_{1}\in \left[0,\omega_{1,\max}\right],\omega_{2}\in \left[0,\omega_{2,\max}\right],\left(\beta_{02}\right)\in \left[0,\beta_{02,\max}\right]$$

where *η*_max_, *ω*_1,max_, *ω*_2,max_ and *β*_02,max_ are the maximums of *η*, *ω*_1_, *ω*_2_ and *β*_02_, respectively.

If we suppose ***σ*** also as a non-informative variable, Equation (13) will become a traditional minimum mean square error (MMSE) estimation problem [32]. In this case, the solution of ***σ*** will suffer from high side lobes and low resolution due to the relatively large aperture gap and short CPI [34], even if *η*, *ω*_1_, *ω*_2_ and *β*_02_ are accurately known, not to mention that they are actually unknown parameters. In order to solve Equation (13) efficiently, here, we exploit the sparse scattering property of the target under the condition of high-frequency scattering, and then, assume the sparse *a priori* distribution of ***σ*** as [22]:

(16) $$f\left(\sigma \right)\propto \prod_{k=1}^{K}\prod_{l=1}^{L}e^{-\frac{2}{q}\left({\left|\sigma_{k,l}\right|}^{q}-1\right)}$$

where *q* ∈ (0,1] is a user parameter. When *q* → 0, the peak of *f*(***σ***) at ***σ*** = 0 becomes infinite. This means that ***σ*** will be equal to zero with a high probability, namely ***σ*** will have a sparse solution in Bayesian inference.

Substituting Equations (14)–(16) into Equation (13) and taking the negative logarithm form of Equation (13), we can obtain:

(17) $$\begin{array}{l} \left(\hat{\sigma },\hat{\eta },{\hat{\omega }}_{1},{\hat{\omega }}_{2},{\hat{\beta }}_{02}\right)=\arg\underset{\sigma ,\eta ,\omega_{1},\omega_{2},\beta_{02}}{\min}C\left(\sigma ,\eta ,\omega_{1},\omega_{2},\beta_{02}\right)\\ \;=\arg\underset{\sigma ,\eta ,\omega_{1},\omega_{2},\beta_{02}}{\min}\left\{\mathrm{MN}\log\eta +\frac{1}{\eta }{\left\|s-A\left(\omega_{1},\omega_{2},\beta_{02}\right)\sigma \right\|}_{2}^{2}+\overset{K}{\underset{k=1}{\text{∑}}}\overset{L}{\underset{l=1}{\text{∑}}}\frac{2}{q}\left({\left|\sigma_{k,l}\right|}^{q}-1\right)\right\} \end{array}$$

where *C* represents the cost function and ‖ · ‖_2_ denotes the Euclidean norm of a vector.

Therefore, based on the MAP criterion and the sparse scattering property of the target, we have converted the T/R-R fusion imaging problem (11) into a joint optimization problem (17). The algorithm of Equation (17) will be discussed in the following section.

## 4. Imaging Algorithm

The purpose of Equation (17) is to find the sparse solution of ***σ***; hence, it is an SSR problem about ***σ***. In the traditional SSR problem, the observing matrix **A** usually is known in advance, and all of the efforts are focused on the recovery of the sparse solution [22]. However, in Equation (17), the observing matrix **A** contains three unknown parameters. According to [25,35], here, we propose a cyclic optimization method to solve Equation (17), which recovers the sparse solution, as well as estimates the parameters in an iterative manner. Firstly, we fix the three parameters, and then, Equation (17) becomes the usual SSR problem, which can be solved by sparse learning via the iterative minimization (SLIM) method proposed in [22]. Secondly, we fix ***σ*** and *η*, and estimate *ω*_1_, *ω*_2_ and *β*_02_ by minimizing the cost function *C*. Then, repeat the two steps until the solution converges. The details are described as follows.

### 4.1. Algorithm Description

The initial values are set as follows: ***σ**ˆ*^0^ is set as traditional range-Doppler (RD) imaging result [10]; ${\hat{\omega }}_{1}^{0}$, ${\hat{\omega }}_{2}^{0}$ and ${\hat{\beta }}_{02}^{0}$ are estimated roughly by imaging geometry and the motion of the target; and ${\hat{\eta }}^{0}=\frac{1}{MN}{\left\|\text{s}\;-\;A^{0}{\hat{\sigma }}^{0}\right\|}_{2}^{2}$. We assume that ***σ***ˆ^i^, *η*ˆ^i^, ${\hat{\omega }}_{1}^{i}$, ${\hat{\omega }}_{2}^{i}$ and ${\hat{\beta }}_{02}^{i}$ are the estimated values after the *i^th^* iteration; then, the (*i* + 1)*^th^* iteration is shown as follows:

Step 1: Sparse Imaging [22]:
  The purpose of this step is to estimate ***σ**ˆ^i^*^+1^ and *ηˆ^i^*^+1^ while fixing ${\hat{\omega }}_{1}^{i}$, ${\hat{\omega }}_{2}^{i}$ and ${\hat{\beta }}_{02}^{i}$. By using these three fixed parameters, the observing matrix ${\text{A}}^{i}\left({\hat{\omega }}_{1}^{i},{\hat{\omega }}_{2}^{i},{\hat{\beta }}_{02}^{i}\right)$ can be determined. Then, the optimization problem (17) becomes:
  (18) $$\left({\hat{\sigma }}^{i+1},{\hat{\eta }}^{i+1}\right)=\arg\underset{\sigma ,\eta }{\min}C\;\left(\sigma ,\eta \right)$$
  which has the same form as the usual SSR problem. It can be solved by the SLIM algorithm [22], which is also a cyclic optimization procedure. When the SLIM algorithm converges, we obtain ***σ**ˆ^i^*^+1^ and *ηˆ^i^*^+1^.
Step 2: Parameter Estimation:
  In this step, we try to estimate ${\hat{\omega }}_{1}^{i+1}$, ${\hat{\omega }}_{2}^{i+1}$ and ${\hat{\beta }}_{02}^{i+1}$ by fixing ***σ**ˆ^i^*^+1^ and *ηˆ^i^*^+1^. Removing the terms which have nothing to do with these three parameters, then the optimization problem (17) becomes:
  (19) $$\begin{array}{ll} \left({\hat{\omega }}_{1}^{i+1},{\hat{\omega }}_{2}^{i+1},{\hat{\beta }}_{02}^{i+1}\right) & =\arg\underset{\omega_{1},\omega_{2},\beta_{02}}{\min}C_{p}\left(\omega_{1},\omega_{2},\beta_{02}\right)\\ & =\arg\underset{\omega_{1},\omega_{2},\beta_{02}}{\min}{\left\|s-A\left(\omega_{1},\omega_{2},\beta_{02}\right){\hat{\sigma }}^{i+1}\right\|}_{2}^{2} \end{array}$$
  where **A** (*ω*_1_, *ω*_2_, *β*_02_) ***σ**ˆ^i^*^+1^ is the estimation of the return signal based on ***σ**ˆ^i^*^+1^. The cost function *C_p_* is the error between **A** (*ω*_1_,*ω*_2_, *β*_02_) ***σ***ˆ*^i^*^+1^ and s. According to our study, *C_p_* (*ω*_1_, *ω*_2_, *β*_02_) is a non-convex function, so we propose a three-dimensional linear search method to solve Equation (19).
  First, we define the discrete search space as Λ:
  (20) $$\Lambda =\left\{\left(\omega_{1},\omega_{2},\beta_{02}\right)|\omega_{1}\in \text{lin}\left(\Omega_{1},{\text{Δ}}_{1}\right),\omega_{2}\in \text{lin}\left(\left(\Omega_{2},{\text{Δ}}_{2}\right),\beta_{02}\in \text{lin}\left(\Omega_{02},{\text{Δ}}_{02}\right)\right)\right\}$$
  where Ω_1_, Ω_2_ and Ω_02_ represent the search range of *ω*_1_, *ω*_2_ and *β*_02_, Δ_1_, Δ_2_ and Δ_02_ are their corresponding step sizes, function lin (Ω, Δ) generates a linear discrete space, whose range is Ω and step size is Δ. Then, by searching the minimum of *C_p_* (*ω*_1_, *ω*_2_, *β*_02_) on A, we will obtain the optimal estimations of these three parameters.
Step 3: Convergence Judgment:
  After Step 1 and Step 2, we can obtain ***σ**ˆ^i^*^+1^, *ηˆ^i^*^+1^, ${\hat{\omega }}_{1}^{i+1}$, ${\hat{\omega }}_{2}^{i+1}$ and ${\hat{\beta }}_{02}^{i+1}$. Then, iteratively operate the two steps until reaching the stop criterion:
  (21) $${\left\|{\hat{\sigma }}^{i+1}-{\hat{\sigma }}^{i}\right\|}_{2}/{\left\|{\hat{\sigma }}^{i+1}\right\|}_{2}<{\text{γ}}_{\sigma }$$
  where *γ_σ_* is a small positive number.

### 4.2. Some Discussions about the Algorithm

In this subsection, we will give some discussions in terms of fast implementation, computational complexity and convergence.

(1) Methods to decrease the computational burden: Because ***σ**ˆ* is a sparse solution, this means that most elements of ***σ**ˆ* are zero or close to zero. Therefore, in Step 2, we only select the large elements of ***σ**ˆ* to calculate the cost function *C*. In this way, the computational burden of Equation (19) will be reduced significantly.
(2) Computational complexity: According to [22] the major computation of Step 1 comes from the matrix vector product (MVP). Obviously, in Step 2, the major computation of Equation (19) is also contributed by the MVP. Therefore, MVP is the main computation of the whole algorithm, and the computational complexity is *O*(*MNKL*).
(3) Convergence of the algorithm: In order to guarantee the convergence of the proposed algorithm, in this paper, we adopt two measures. First, the SLIM algorithm [22] is exploited in Step 1. SLIM belongs to the Bayesian compressive sensing (CS) method, which is relatively robust against noise. Second, in Step 2, only the strong scatterers are selected to calculate the cost function C, which can reduce the impact of noise on the convergence of the algorithm to a certain degree. Although, it is difficult to prove the convergence of the algorithm directly. However, by the above two measures, we can ensure that the cost function will decrease after every iteration. Moreover, according to the simulation results, usually the cost function will converge after only a few iterations.

## 5. Simulation Results

**Figure 4.**
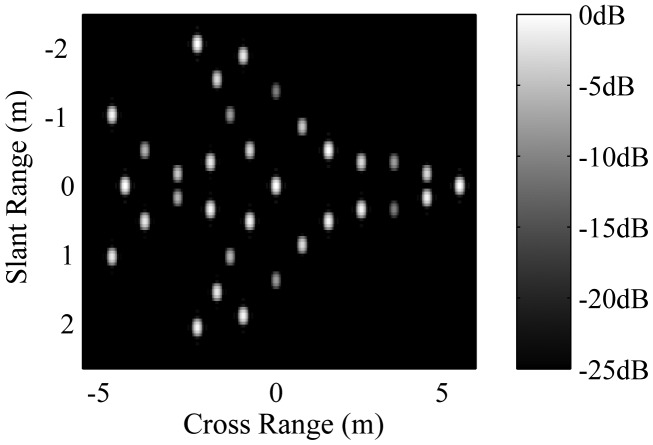
Target model.

In this section, we demonstrate some simulation results to verify the effectiveness of the proposed T/R-R fusion imaging method. The simulation conditions are as follows: the target shown in Figure 4 is modeled as a set of dominant point scatterers with different scattering coefficients. During the short CPI, it is moving along the *x*-axis at the speed of *v* = 300 *m/s.* Assume that the bistatic angle is *β*_02_ = 4.3° at *t_m_* = 0 and the distances between the target and Radar 1 and Radar 2 are *R*_1_ (0) = 10 km and *R*_2_ (0) = 12 km, respectively. Then, according to the theory of ISAR and B-ISAR, we can calculate that the truth-values of the rotational speeds are *ω*_1_ = 0.030 rad/s and *ω*_2_ = 0.025 rad/s. The signal-to-noise ratio (SNR) of the return signal is 15 dB. More details about the simulation conditions are given in Table 1, and the user parameter *q* in Equation (16) is chosen as 0.6 (in fact, we found not much of a difference of the various values of *q* < 1). In the simulation, the initial values of *ω*_1_, *ω*_2_ and *β*_02_ are set to 0.04 rad/s, 0.04 rad/s and 6°, respectively.

Before imaging simulation, we calculate the theoretical resolutions of Radar 1, Radar 2 and the T/R-R fusion system according to the theory of ISAR and B-ISAR. In the slant range direction, the resolutions are determined by the bandwidth of the transmitted signal and the bistatic angle, as shown in Table 2. Because *β*_02_ = 4.3° is small, they have approximately the same slant range resolutions as 0.38 m. The cross-range resolutions are inversely proportional to the sizes of the apertures Δ*θ*_1_, Δ*θ*_2_ and Δ*θ*. These apertures can be obtained by substituting the truth-values of *ω*_1_, *ω*_2_ and *β*_02_ into Equations (9) and (10), where *T_m_* = 0.25 s. Then, the cross-range resolutions are calculated and shown in Table 2, where A = 0.03 m is the wavelength corresponding to the carrier frequency. We can see that if Radar 1 and Radar 2 are considered separately, their cross-range resolutions are 2.00 m and 2.20 m, respectively, which are very poor compared to their slant range resolutions. The proposed T/R-R fusion imaging system constructed a large spanned aperture with the size being 2.56°, which is much larger than Δ*θ*_1_ and Δ*θ*_2_. Thus, the cross-range resolution is improved to 0.34 m, which is more than five-times better than either Radar 1 or Radar 2.

### 5.1. Imaging Simulation

**Figure 5.**
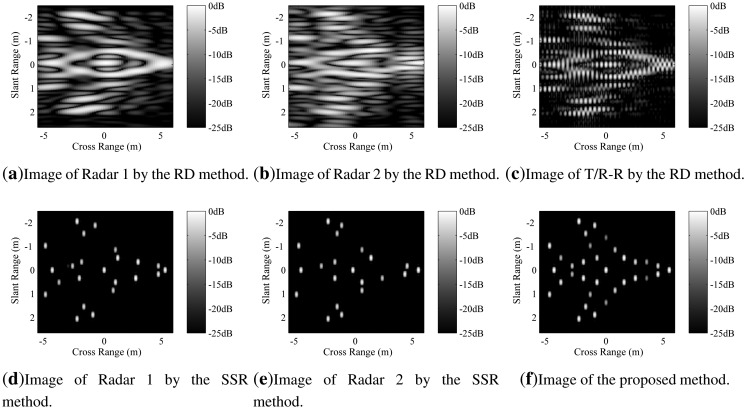
Imaging results. In (**a**)-(**e**), the parameters *ω*_1_, *ω*_2_ and *β*_02_ are set to be the truth-values in advance, because the range-Doppler (RD) method or the sparse signal recovery (SSR) method with the short coherent processing interval (CPI) does not have the ability to estimate the parameters accurately, while in (f), the parameters are estimated during the imaging iteration by our method.

The proposed sparse fusion imaging method is utilized to reconstruct the image of the target. For the purpose of comparison, the images obtained by Radar 1 and Radar 2 separately are also given. Furthermore, all of these three kinds of images are reconstructed both by the traditional RD method and the SSR method. The imaging results are shown in Figure 5.

Figure 5a shows the image of Radar 1 by the RD method. One can see that in slant range direction, it has the ability to distinguish the point scatterers, because of the sufficient signal bandwidth. However, in cross-range direction, its resolution is too poor to distinguish the point scatterers. Figure 5d is the SSR result corresponding to Figure 5a. In this figure, only a small part of the point scatterers are recovered with accurate locations and amplitudes; many scatterers are recovered with errors in locations or amplitudes, and the others are missed. That is because the aperture of Radar 1 is so small that even the SSR method cannot recover the image correctly. The images of Radar 2 obtained by the RD and SSR method are shown as Figure 5b,e, respectively. Similar to Radar 1, neither of the methods can reconstruct the image correctly, due to the small aperture. Figure 5c shows the image obtained by directly solving the fusion model (11) with the RD method, where the parameters are set to be their truth-values. It shows that although the cross-range resolution is enough to distinguish the point scatterers; the image suffers from very high sidelobes, due to the large aperture gap between the two sub-apertures. Finally, the image obtained by the proposed sparse fusion imaging method is shown in Figure 5f. Compared to the true target model shown in Figure 4, we can see that the image is well focused with high resolution and low side lobes; both the locations and amplitudes of all the scatterers are recovered correctly. Therefore, it proves that our method has the ability to reconstruct a high resolution image with a very short CPI time.

### 5.2. Algorithm Performance Simulation

**Figure 6.**
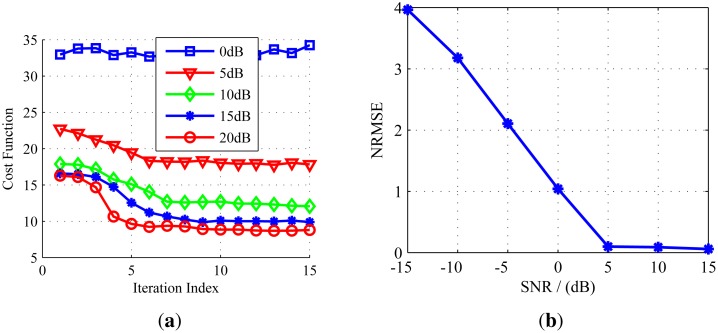
(**a**) Convergence performance of the algorithm under different SNR; (**b**) normalized root mean square error (NRMSE) of the images *versus* SNR.

The convergence, as well as the parameter estimation performance of the algorithm are assessed by 60 Monte-Carlo trials. Figure 6a shows the convergence performance of the cost function under different SNR conditions. We can see that when SNR = 0 dB, the cost function cannot converge, due to the strong noise, but when SNR ≥ 5 dB, the cost function will converge after only a few iterations. Moreover, with the increase of the SNR, the convergence rate becomes faster. In order to represent the recovery performance of the proposed method under different SNR conditions, we define the normalized root mean square error (NRMSE) between the imaging result and the original target model as follows:

(22) $$\text{NRMSE}={{\left\|{\hat{\sigma }}_{m}-\sigma \right\|}_{2}/\left\|\sigma \right\|}_{2}$$

where ‖• ‖_2_ denotes the Euclidean norm, ***σ*** is the scattering coefficient vector of the target model and ***σ**ˆ*_m_ is the average estimation of ***σ*** over 60 Monte-Carlo trials. NRMSE demonstrates the recovery performance of the imaging method; a smaller NRMSE means better recovery performance. Figure 6b shows the values of NRMSE *versus* SNR. It shows that with the increase of the SNR, the values of NRMSE decrease, *i.e.*, the recovery performance becomes better. When SNR ≥ 5 dB, the NRMSE is close to zero, which means that under this condition, the imaging result is substantially the same as the original target model.

**Figure 7.**
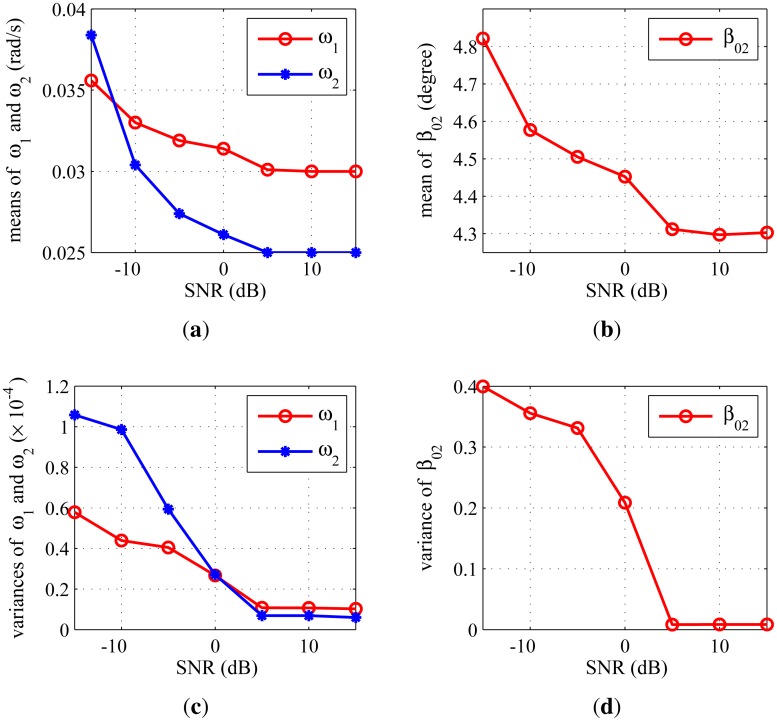
Parameter estimation accuracy *versus* SNR: (a) means of *ω*_1_ and *ω*_2_; (**b**) mean of *β*_02_; (**c**) variances of *ω*_1_ and *ω*_2_; (**d**) variance of *β*_02_.

Figure 7 shows the parameter estimation accuracy, Figure 7a,b is the means of *ω*_1_, *ω*_2_ and *β*_02_*versus* SNR, and Figure 7c,d is their variances. It shows that when SNR ≥ 5 dB, the average estimations of *ω*_1_, *ω*_2_ and *β*_02_ are very close to their truth-values: 0.030 rad/s, 0.025 rad/s and 4.3°, respectively, and the variances of the estimations are fairly small. This indicates that the proposed method has high parameter estimation accuracy.

## 6. Conclusions

This paper has proposed a sparsity-based fusing imaging method for a moving target in T/R-R configuration. Based on the idea of coherently fusing two widely separated apertures into a large aperture and by exploiting the sparse scattering property of the target, the cross-range resolution of the image is improved significantly. The details of the method, as well as its corresponding algorithm are presented. We also give some comments on the fast implementation and the convergence of the algorithm. Finally, some simulation results are given to validate the proposed method. They show that the proposed sparse fusion imaging method has the ability to reconstruct a high resolution image with a very short CPI time, and the algorithm can converge within a few iterations.

## Figures and Tables

**Table 1.** Simulation Conditions.

**System Parameter**	**Value**
Carrier frequency of the signal *f*_0_	10 GHz
Bandwidth of the transmitted signal *B*	400 MHz
Pulse repetition frequency *PRF*	80 Hz
Numbers of samples of fast-time and slow-time	*N* = 30 *M* = 20
Image size	*Length* = 12 m, *Width* = 6 m
Separations between cross- and slant range bins	*dx* = 0.2 m, *dy* = 0.2 m
Numbers of cross- and slant range bins	*K* = 60, *L* = 30

**Table 2.** Aperture sizes and theoretical resolutions.

	**Slant Range Resolution**	**Aperture Size**	**Cross-Range Resolution**
Radar 1	$\rho_{sr,1}=\frac{c}{2B}=0.38\text{m}$	Δ*θ*_1_ = 0.43°	$\rho_{cr,1}=\frac{\lambda }{2\Delta \theta_{1}}=2.00\text{m}$
Radar 2	$\rho_{sr,2}=\frac{c}{2B\cos^{2}\left(\beta_{02}/2\right)}\approx 0.38\text{m}$	Δ*θ*_2_ = 0.39°	$\rho_{cr,2}=\frac{\lambda }{2\Delta \theta_{2}}=2.20\text{m}$
T/R-R	$\rho_{sr}=\frac{c}{2B\cos^{2}\left(\beta_{02}/4\right)}\approx 0.38\text{m}$	Δ*θ* = 2.56°	$\rho_{cr}=\frac{\lambda }{2\Delta \theta }=0.34\text{m}$
